# Supplementary material for: Encapsulation of Luminescent Gold Nanoclusters into Synthetic Vesicles
Source: Nanomaterials (Basel). 2022 Nov 2;12(21):3875. doi: 10.3390/nano12213875 (PMC9655092; doi:10.3390/nano12213875)
Supplement: Supplementary file 1 [file nanomaterials-12-03875-s001.zip › nanomaterials-1983666-supplementary-conversion/nanomaterials-1983666-supplementary-conversion.pdf]

nanomaterials

Supplementary Materials

Encapsulation of Luminescent Gold Nanoclusters into Synthetic Vesicles

Regina M. Chiechio [1,2,3], Solène Ducarre [3], Célia Marets [1], Aurélien Dupont [4], Pascale Even-Hernandez [1], Xavier Pinson [5], Stéphanie Dutertre [5], Franck Artzner [6], Paolo Musumeci [2], Célia Ravel [7,8], Maria Jose Lo Faro [2,3] and Valérie Marchi [1,*]

[1] Institut des Sciences Chimiques de Rennes, CNRS UMR 6226, Université Rennes 1, F-35000 Rennes, France
[2] Dipartimento di Fisica e Astronomia "Ettore Majorana", Università Di Catania, Via Santa Sofia 64, 95123 Catania, Italy
[3] IMM-CNR, Via S. Sofia 64, 95123 Catania, Italy
[4] BIOSIT, Inserm, CNRS UMS 3480, Université Rennes1, US_S 018, F-35000 Rennes, France
[5] Microscopy Rennes Imaging Centre, SFR Biosit, CNRS UMS 3480—US INSERM 018, Université Rennes 1, F-35000 Rennes, France
[6] Institut de Physique, CNRS UMR 6251, Université Rennes 1, F-35000 Rennes, France
[7] Service de Biologie de la Reproduction-CECOS, CHU Rennes, 35000 Rennes, France
[8] Irset (Institut de Recherche en Santé, Environnement et Travail), Inserm, EHESP, Université Rennes 1, F-35000 Rennes, France
* Correspondence: valerie.marchi@univ-rennes1.fr

Citation: Chiechio, R.M.; Ducarre, S.; Marets, C.; Dupont, A.; Even-Hernandez, P.; Pinson, X.; Dutertre, S.; Artzner, F.; Musumeci, P.; Ravel, C.; et al. Encapsulation of Luminescent Gold Nanoclusters into Synthetic Vesicles. *Nanomaterials* **2022**, *12*, x. https://doi.org/10.3390/xxxxx

Academic Editor: Rosario Pereiro

Received: 6 October 2022
Accepted: 26 October 2022
Published: 2 November 2022

Publisher's Note: MDPI stays neutral with regard to jurisdictional claims in published maps and institutional affiliations.

Copyright: © 2022 by the authors. Submitted for possible open access publication under the terms and conditions of the Creative Commons Attribution (CC BY) license (https://creativecommons.org/licenses/by/4.0/).

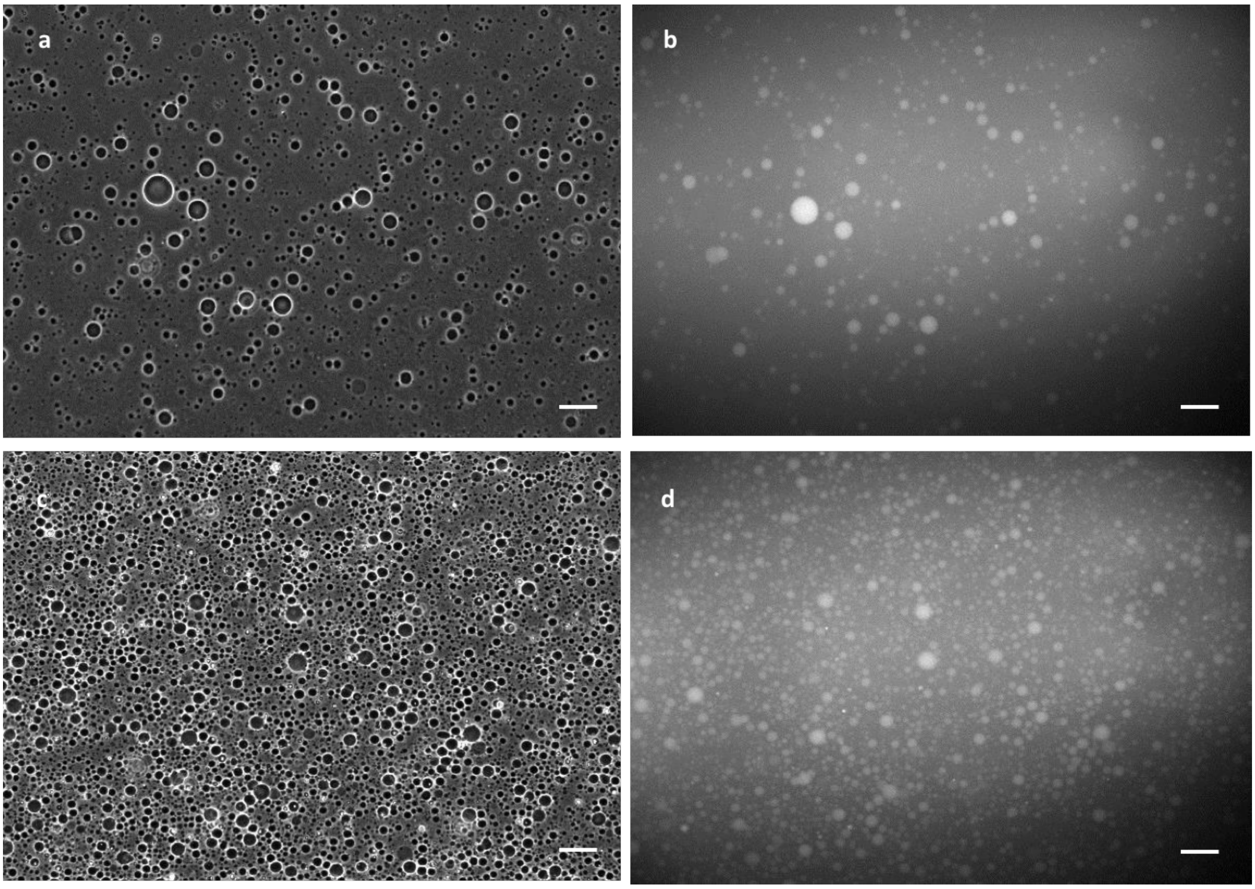

Figure S1. Phase contrast (left) or Fluorescence (right) Optical microscope images of C_3E_6D Red NCs encapsulated inside: (**a,b)** DOPC and (**c,d)** DOPC/DOTAP GUVs. Scale bar 20 μm.

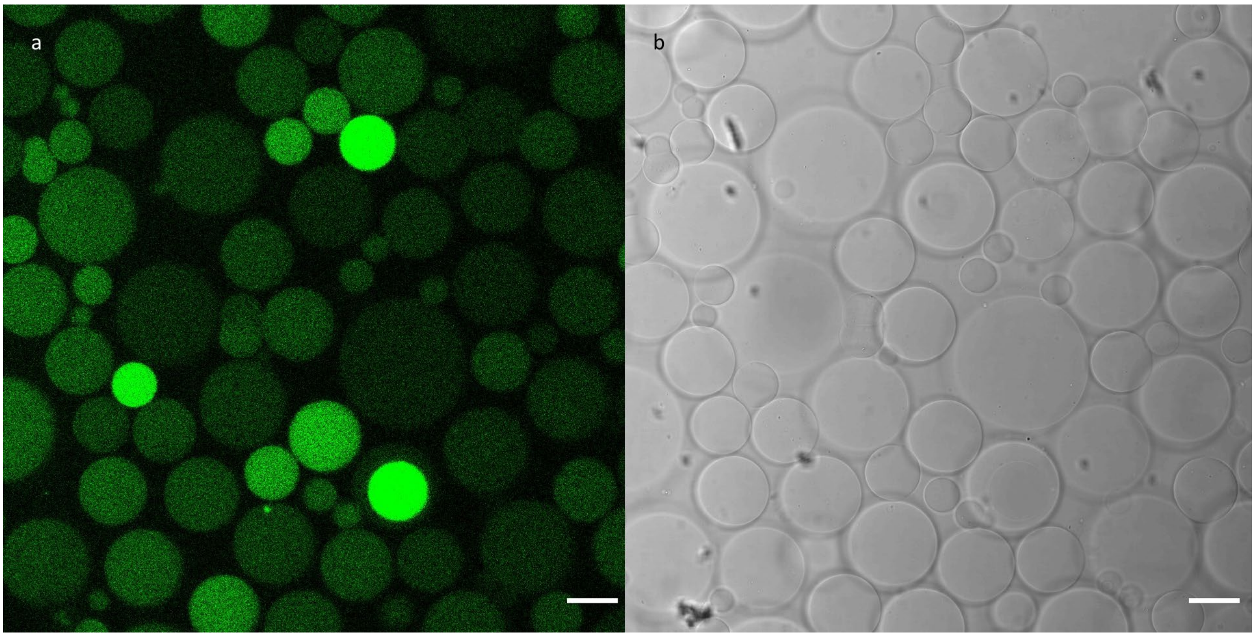

Figure S2. Confocal microscope (**a**) fluorescence and (**b**) bright field images of C_3E_6D Blue NCs encapsulated inside DOPC GUVs. Scale bar 20 μm.

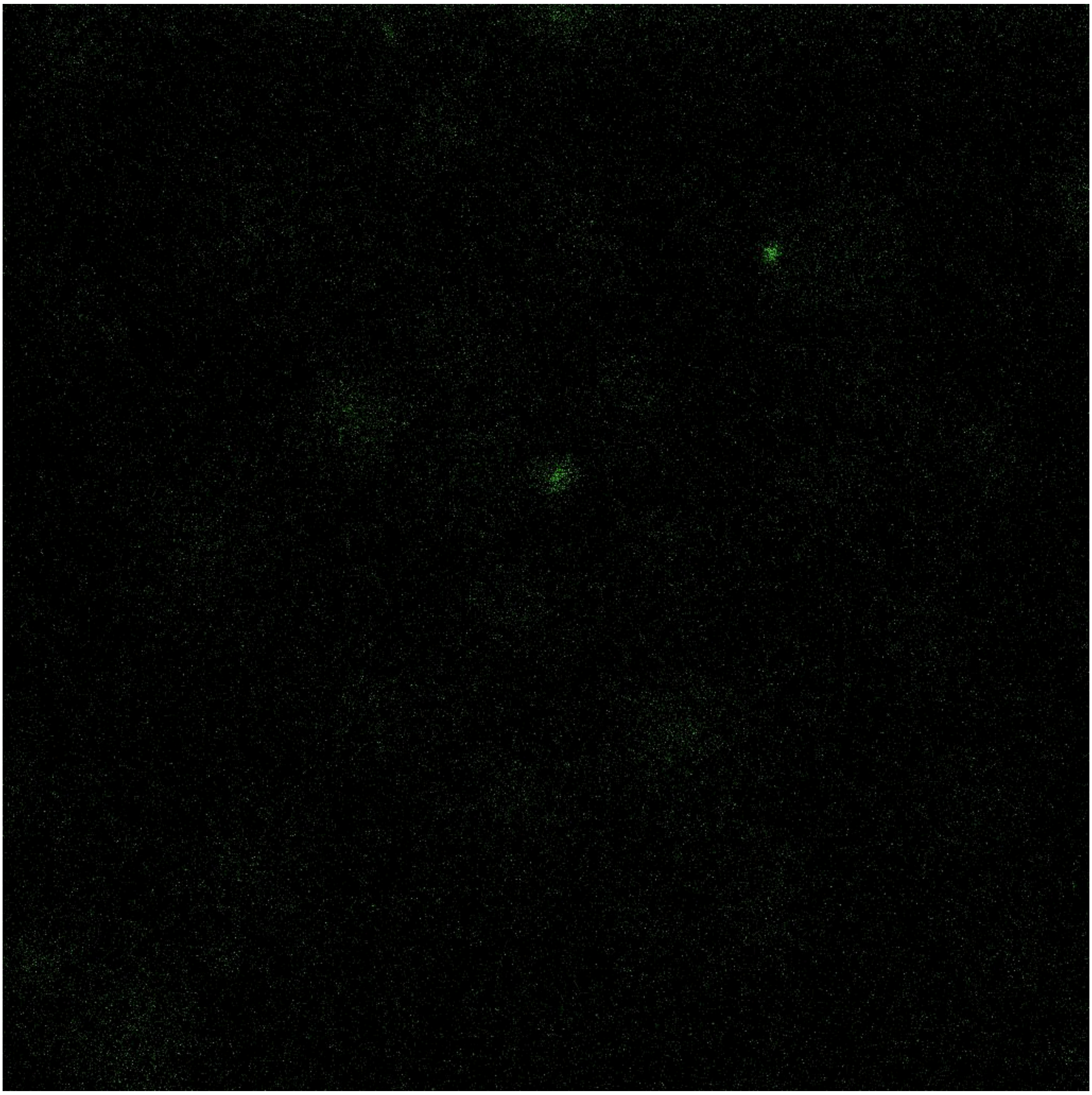

Figure S3. Confocal microscopy images (in z-stack) of C_3E_6D Blue NCs encapsulated inside DOPC GUVs corresponding to Figure **S2a**.

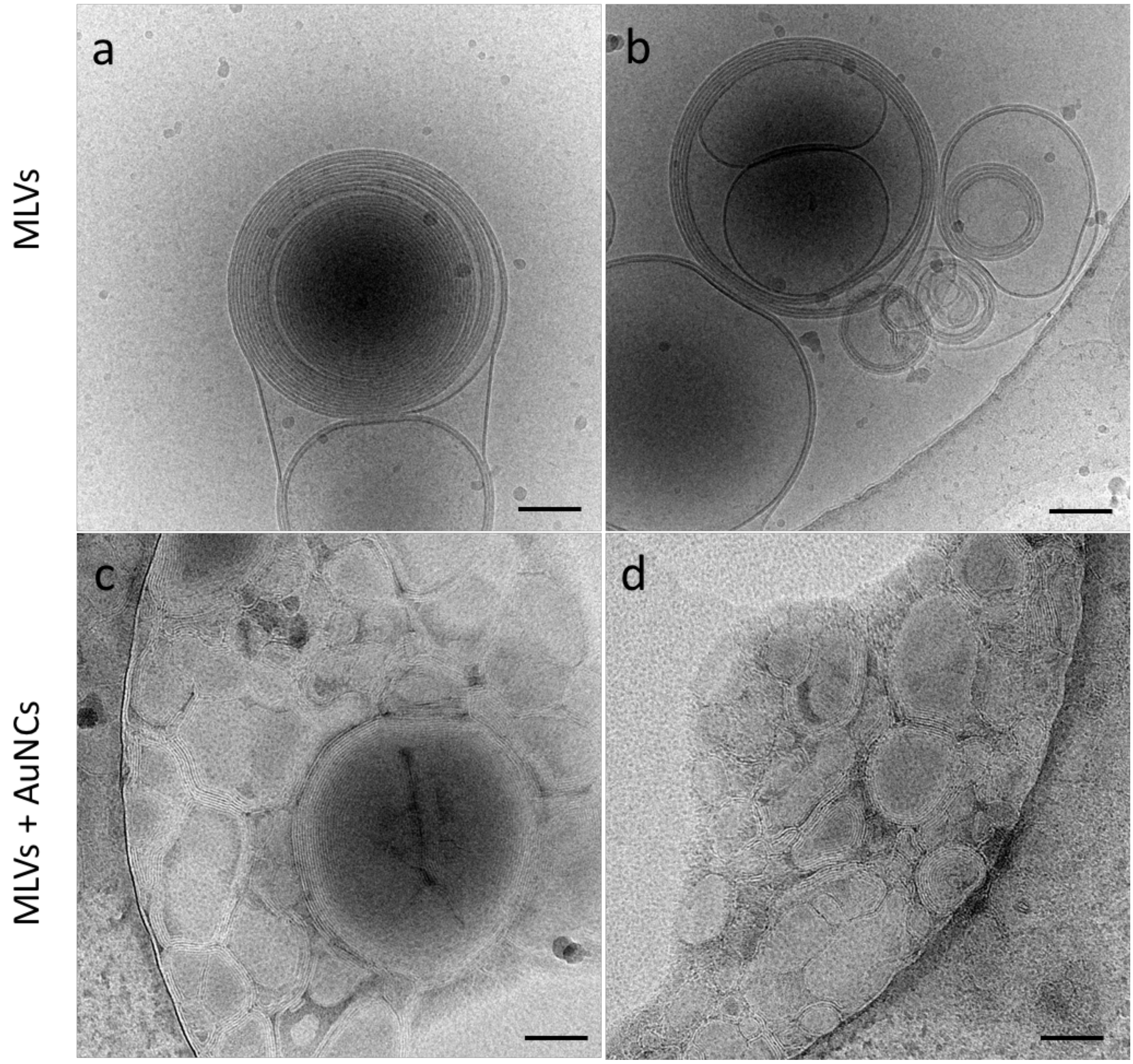

Figure S4. Cryo-TEM images of DOPC MLVs alone (**a**,**b**) and with Au NCs (**c**,**d**). Scale bar 200 nm.

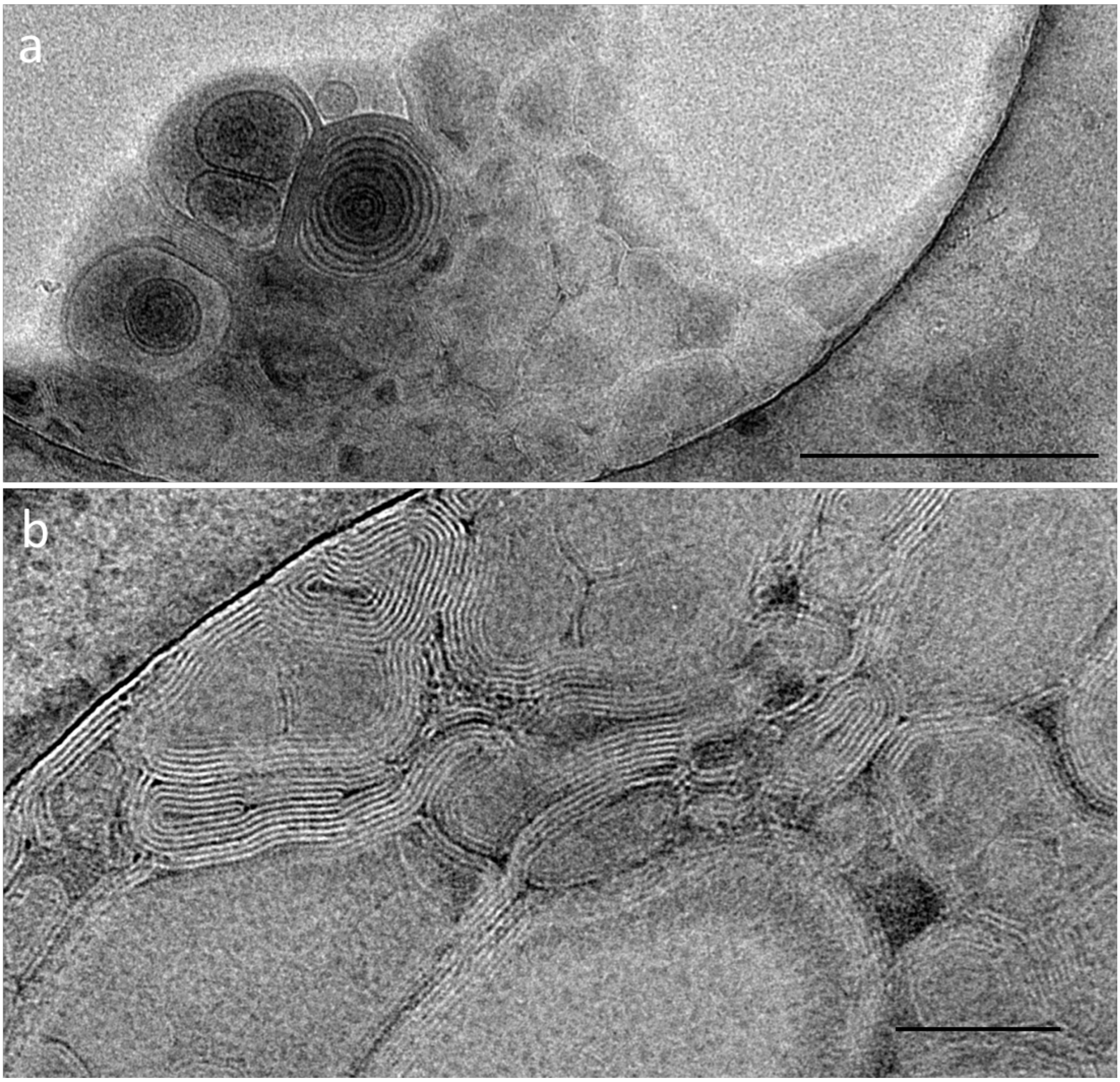

Figure S5. Cryo-TEM images of DOPC MLVs with Au NCs (**a**,**b**). Scale bar 400 nm.

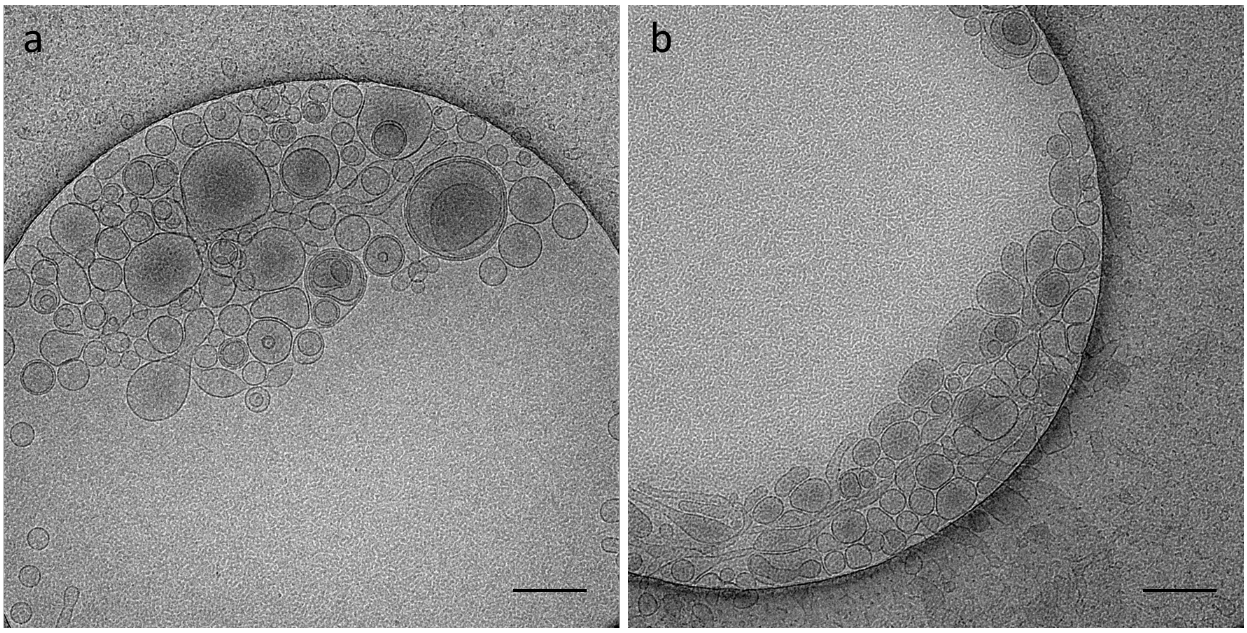

Figure S6. Cryo-TEM images of DOPC LUVs alone (**a**,**b**). Scale bar 200 nm.

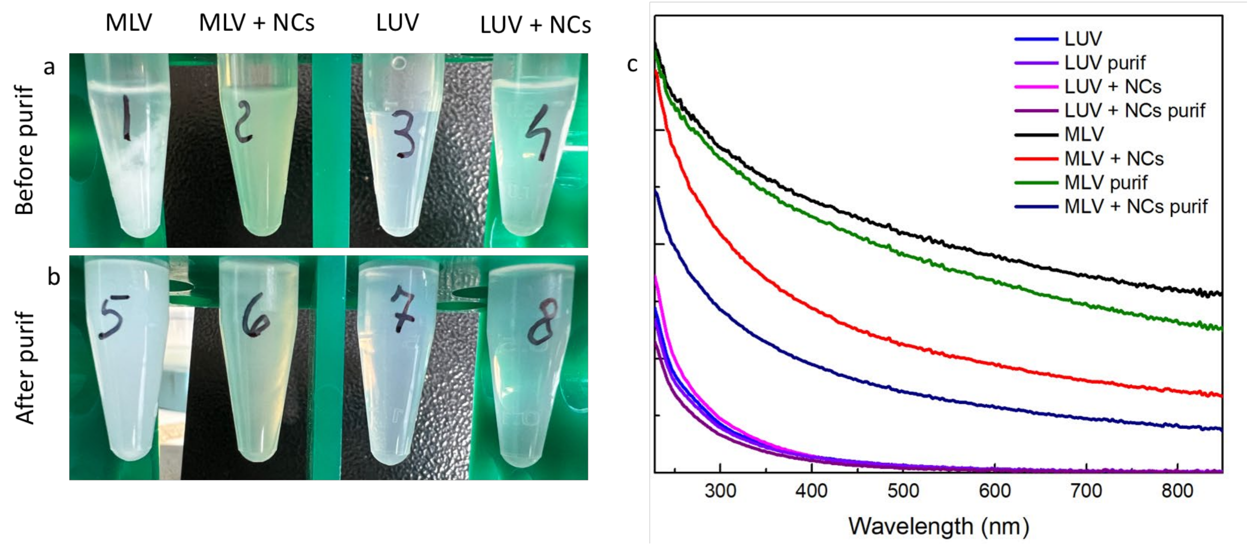

Figure S7. (**a**,**b**) Images of suspensions composed of the multilamellar vesicles (MLV) or unilamellar vesicles (LUV) alone and in presence of red AuNCs before and after purification; (**c**) corresponding UV-Vis spectra of the vesicles suspensions.
